# Profile of Risk Factors for Non-Communicable Diseases in Punjab, Northern India: Results of a State-Wide STEPS Survey

**DOI:** 10.1371/journal.pone.0157705

**Published:** 2016-07-07

**Authors:** J. S. Thakur, Gursimer Jeet, Arnab Pal, Shavinder Singh, Amarjit Singh, S. S. Deepti, Mohan Lal, Sanjay Gupta, Rajender Prasad, Sanjay Jain, Rajiv Saran

**Affiliations:** 1 School of Public Health, Post Graduate Institute of Medical Education and Research, Chandigarh, India; 2 Department of Biochemistry, Post Graduate Institute of Medical Education and Research, Chandigarh, India; 3 Department of Community Medicine, Christian Medical College, Ludhiana, India; 4 Department of Community Medicine, Government Medical College, Patiala, India; 5 Department of Community Medicine, Government Medical College, Amritsar, India; 6 Department of Community Medicine, Guru Gobind Singh Medical College, Faridkot, India; 7 Department of Internal Medicine, Post Graduate Institute of Medical Education and Research, Chandigarh, India; 8 Department of Internal Medicine and Epidemiology, University of Michigan, Ann Arbor, Michigan, United States of America; Iran University of Medical Sciences, ISLAMIC REPUBLIC OF IRAN

## Abstract

**Background:**

Efforts to assess the burden of non-communicable diseases risk factors has improved in low and middle-income countries after political declaration of UN High Level Meeting on NCDs. However, lack of reliable estimates of risk factors distribution are leading to delay in implementation of evidence based interventions in states of India.

**Methods:**

A STEPS Survey, comprising all the three steps for assessment of risk factors of NCDs, was conducted in Punjab state during 2014–15. A statewide multistage sample of 5,127 residents, aged 18–69 years, was taken. STEPS questionnaire version 3.1 was used to collect information on behavioral risk factors, followed by physical measurements and blood and urine sampling for biochemical profile.

**Results:**

Tobacco and alcohol consumption were observed in 11.3% (20% men and 0.9% women) and 15% (27% men and 0.3% women) of the population, respectively. Low levels of physical activity were recorded among 31% (95% CI: 26.7–35.5) of the participants. The prevalence of overweight and obesity was 28.6% (95% CI: 26.3–30.9) and 12.8% (95% CI: 11.2–14.4) respectively. Central obesity was higher among women (69.3%, 95% CI: 66.5–72.0) than men (49.5%, 95% CI: 45.3–53.7). Prevalence of hypertension in population was 40.1% (95% CI: 37.3–43.0). The mean sodium intake in grams per day for the population was 7.4 gms (95% CI: 7.2–7.7). The prevalence of diabetes (hyperglycemia), hypertriglyceridemia and hypercholesterolemia was 14.3% (95% CI: 11.7–16.8), 21.6% (95% CI: 18.5–25.1) and 16.1% (95% CI: 13.1–19.2), respectively. In addition, 7% of the population aged 40–69 years had a cardiovascular risk of ≥ 30% over a period of next 10 years.

**Conclusion:**

We report high prevalence of risk factors of chronic non-communicable diseases among adults in Punjab. There is an urgent need to implement population, individual and programme wide prevention and control interventions to lower the serious consequences of NCDs.

## Introduction

With introduction of the National Health Mission in 2005, India has been rapidly progressing towards attainment of good health indicators with fall in levels of infant and maternal mortality and trend reversal regarding HIV, malaria, and tuberculosis.[1] However mortality (60% of total deaths) and morbidity burden due to NCDs is increasing.[2] Government of India launched a new health programme, “National Program on Prevention and Control of Cancer, Diabetes, Cardiovascular Diseases and Stroke (NPCDCS) with renewed focus on major NCD’s to energize the multisectoral and multilevel response from health systems and public health authorities.

The Punjab is a large state in northern India (population of 2.7 million as per 2011 census) which enjoys the reputation of being the ‘food basket’ of nation with a medium human development level.[3] It is witnessing a disturbing growth in NCDs related mortality.[4] As part of national efforts to control NCDs, NPCDCS program was introduced in Punjab State as well. However designing of effective interventions and subsequent assessment of benefits of program implementation required good quality evidence as base line estimates. No organized studies have been conducted in the state in the past to assess prevalence of NCD risk factors, though surveys have been conducted to assess burden of individual risk factors.[5–7] Contemporary state-specific data, so crucial for ongoing evidence-based public health interventions, was therefore, lacking. This lack of quality health information, essential for planning and implementing policies for effective disease control and health improvement was realized and Risk Factors survey was planned. Survey generated state-specific NCD risk factors data using WHO STEP wise approach to Surveillance (STEPS) [8] to inform health policy and generate estimates for National NCD Monitoring framework indicators.[9] In addition survey implementers aimed at building capacity of medical schools in the state to undertake such type of surveys.

## Methods

STEPS Survey Punjab was undertaken in 2014–2015 over a period of 15 months with 8 months of data collection for survey. Five medical institutes in the state participated in implementation with Post Graduate Institute of Medical Education and Research, Chandigarh acting as coordinating cum implementing agency. The survey adopted a multistage, geographically clustered, probability-based sampling approach using the 2011 census as sampling frame.

The sample size was calculated based on the previous estimates of prevalence of physical inactivity as around 50%.[10, 11] 95% Confidence Intervals and the corresponding margin of error of 0.05 were used as recommended by STEPS manual.[8] The total sample size estimate (obtained by summing across the age/sex/residence strata) was adjusted for the design effect (1.5). Using these values a sample size of 4609 was derived which was adequate to provide state wide results by two age groups (18–44, 45–69), both sexes (male, female) and residence (urban, rural). Assuming a response rate of 85% [12], sample size was raised to 5400 for this study.

A proportionate allocation as per census distribution in urban and rural area was adopted. A list of 2541 wards and 12006 villages comprised our sampling frame. In urban areas, a three-stage procedure was followed. In the first stage, wards were selected with Population Proportional to Sample size. In the second stage, one Census Enumeration Block (CEB) was randomly selected from each sampled ward. In the final stage, households were randomly selected within each CEB using the systematic random sampling procedure. Similar process was followed for villages. However for rural areas, a two stage procedure was followed. Village was considered as primary sampling unit (PSU). From each selected PSU in rural area and from each selected Census Enumeration Block (CEB) in urban area, 54 households were selected. The ultimate sampling units were the households and one individual in the age group of 18–69 years residing in the selected household was selected using the Kish method.[13, 14]

The STEPS questionnaire (version 3.1) was used with local adaptations. Translation to Punjabi and back translation into English was done before using the translated versions. Information was collected using traditional paper pen methods. Socio demographic and behavioral information was collected in Step 1. Study tool covered several different domains. This article gives description of results for modules on tobacco use, alcohol consumption, diet and dietary salt, physical activity, health screening, past history of hypertension, diabetes, cardiovascular diseases and treatment being taken. For Physical measurements (Step 2), in addition to blood pressure measurements and Body Mass Index calculation (height, weight), waist circumference was measured because of population’s predisposition to central obesity.[15, 16] Biochemical tests were conducted to measure fasting blood glucose, total cholesterol, triglycerides and urinary sodium levels in Step 3.

### Data collection (STEP 1)

Interviewers were field investigators having post graduate educational background in sociology/psychology/anthropology (for interviews/ physical measurements) and bachelor/ master’s degree (BSc/ MSc) in medical laboratory (for sample collection and processing). They were selected from a cross section of persons who had experience working in health or participated in previous population censuses and /or conducting other surveys. All the recruited staff underwent training for four days which covered all the three steps with classroom interactive sessions, skill development and field visits for pilot testing of the instrument for Step 1 and 2.

Using maps and addresses of households selected, a starting point was determined randomly and thereafter, every selected household, depending on the number of households within the village/ urban area were surveyed. If no one was present in the selected household, a notification of visit was left and the revisit was made up to 3 times at timings suitable to participant. All the three steps were conducted at households of the participants.

Demographic information included income, education and occupation in addition to basic information on sex, age, residential area (urban/rural) and contact number. Information on tobacco consumption for smoking as well as smokeless tobacco was collected. Knowledge, attitude and practices regarding fruit, vegetable intake and dietary salt were assessed. For alcohol, information was collected to capture amount, frequency as well as patterns of drinking. Physical activity information was collected using GPAQ format (as part of STEPS tool) and information was asked in all the three domains i.e. work, transport and leisure, which was further classified into low (less than 600 MET minutes per week), moderate (600–1500 MET minutes per week) and high (more than 1500 MET minutes per week).

In order to identify the reach of screening, diagnostic and treatment services, prior history of screening, diagnosis for NCDs or NCD risk factors (diabetes, hypertension, cardiovascular diseases) and treatment being taken was asked. For treatment use of traditional systems for NCDs was also asked as they are recognized systems of medicine in India and are collocated with allopathic medicine facilities under public health system. To measure the reach of health promotion activities by health worker or physician, questions were asked for advice received pertaining to life style modifications.

### Physical measurements (STEP 2)

Height, weight, waist circumference were measured using standardised instruments recommended by WHO STEPS while conducting such community based surveys (SECA, GmbH, Hamburg, Germany). Instruments were calibrated routinely during the survey. Height and weight of participants were measured while they were barefoot with light clothing. Weight was measured to the nearest 10gms using an electronic scale, while height was measured to the nearest 0.1 cm using a portable stadiometer. Body Mass Index was calculated as weight in Kilograms/ height in meters squared. For blood pressure measurement, electronic equipment (OMRON HEM 7120, Omron Corporation, Kyoto, Japan) was used that has been recommended. Three blood pressure measurements were undertaken at three minutes interval in a seated position, and average of last two readings was taken.

### Biochemical measurements (STEP 3)

Written instructions regarding fasting, appointment date for blood test were given to the participant for STEP 3 on first visit. Only alternate individual (50%) of the initial sample was subject to biochemical measurements. A mix of dry and wet chemistry methods was used to assess biochemical profile of participants using blood and urine samples. For blood glucose, dry chemistry method was used by capillary blood glucose measurement device (Optium H, Freestyle). For lipid profile i.e. cholesterol and triglycerides measurements, blood samples were drawn on individuals after 10–12 hours fasting. 5 ml of venous blood was taken in sitting position, and centrifuged immediately to separate serum, and transferred under cold chain condition to the Central Reference Laboratory of Department of Biochemistry (Post Graduate Institute of Medical Education and Research, Chandigarh, India). Spot urine sample was taken from participants at the time of STEP 3, for urinary analysis to detect presence of protein and creatinine using a point of care device called U CHEK (Biosense Technology). For urinary sodium, 5 ml was stored in a separate aliquot and transferred along with serum samples to laboratory at PGIMER, Chandigarh.

Serum total cholesterol was determined enzymatically by using cholesterol esterase (CE) and cholesterol oxidase (CO). Serum triglycerides were estimated by a method based on using a lipoprotein lipase. Laboratory measurements of lipids (total cholesterol and triglycerides), were made on Modular P 800 autoanalyzer (Roche Diagnostics, Germany) using commercially available kits (Roche Diagnostics, Germany). Urinary sodium was measured on indirect ISE 900 module attached to Modular P 800 autoanalyzer (Roche Diagnostics, Germany).

The Institute Ethical Committee of Post Graduate Institute of Medical Education and Research, Chandigarh, and the Technical Advisory Committee of the survey approved the study protocol and reviewed the implementation of survey. Informed and written consent was taken from all participants.

### Definitions used

Cut off values recommended by STEPS were used for prevalence estimation.[8] Current smoking (smoked in the past 30 days) and harmful alcohol use ((i.e. ≥ 60 g of pure alcohol for men and ≥ 40 g of pure alcohol for women on an average day) was considered a NCD risk factor. Individuals who consumed less than five servings of fruits and vegetables per day were considered as ‘at risk’ group. Overweight was defined as BMI between 25–29.9 kg /m^2^ and obesity as ≥30 kg/m^2^. Abdominal obesity was defined as a waist circumference of ≥90 cm in men and ≥80 cm in women. Hypertension was defined as a systolic blood pressure of ≥140 mm of Hg, or a diastolic blood pressure of ≥90 mm of Hg or the use of blood pressure-lowering medications for hypertension. Individuals with fasting capillary blood glucose of ≥110 mg /dl or on medications for high blood sugar were considered to have diabetes mellitus. A raised serum cholesterol level was defined as total cholesterol ≥190 mg/dl, whereas hypertriglyceridemia was defined as a serum triglyceride value ≥150 mg/dl. Similarly raised intake of sodium was defined as salt intake of more than 5gm per day.

### Statistical methods

Considering the unequal distribution of age, sex and residence in the population, appropriate weights were used for all data analyses. Due to variations in the sample between different steps, separate weights were calculated for all the 3 different steps. The overall weight for each individual comprised of all three weights i.e. sample weight (inverse of the probability of selection for the participant), non-response weight (non-response rate for participant's age/sex group) and population weight (adjustment for participant's age/sex group) multiplied together. Data cleaning as well as data analysis was done using Epi info version 3.5.2. All estimates are presented with 95% confidence intervals (CIs), significance of difference in results between different groups was observed by comparing 95% CIs, which were calculated using Taylor series linearization. Data were analyzed by age group, sex, and residence. Prevalence of different risk factors and proportion above acceptable levels was determined. In addition mean values of relevant behavioural risk factors and other continuous variables such as body mass index (BMI), waist circumference, blood pressure, and different biochemical variables were determined. Multiple logistic regression models were constructed relating the behavioural risk factors (tobacco, alcohol, physical activity), physical measurements (obesity, hypertension) and biochemical risk factors (hyperglycemia, hypercholesterolemia), as dependent variables which were modelled individually to the demographic variables (predictor) i.e. age, sex, gender, occupation, marital status (modelled simultaneously). Odds Ratios calculated using the model reflected the risk of having NCD risk factor in the selected group of participants against the risk in the reference group.

## Results

Overall response rate (multiple of household and individual level response rates) for STEP 1, STEP 2 and STEP 3 of the survey were 95%, 95% and 93% respectively. Of 5400, a total of 5312 households responded, of which a total of 5127 individuals gave consent for STEP 1 and 2. Similarly for STEP 3, out of 2700 households contacted, 2660 responded and a total of 2499 individuals from these households gave consent to blood and urine sampling.

A total of 5127 participants (54% women and 46% men) stratified by age group, sex and place of residence were included. Blood and urine samples (5ml each) were collected from 2499 participants (64% women, 33% men). 65% of the study participants were in the 18–44 year age group. Urban-rural distribution of study participants was found to be similar to urban rural proportion in the state population. About 40% of the participants (2067) were home makers followed by self-employed (1290, 25%) and non-government employees (957, 19%). Details of study sample characteristics are given in Table 1.

**Table 1:** Socio-demographic profile of study participants in STEPS Survey, Punjab, India*.

		Male (N = 2381)	Females (N = 2746)	Both Sexes (N = 5127)
		n (%)	n (%)	n (%)
**Age**	18–44	1512 (64)	1832 (67)	3344 (65)
	45–69	869 (36)	914 (33)	1783 (35)
**Residence**	Rural	1438 (60)	1658 (60)	3096 (60)
	Urban	943 (40)	1088 (40)	2031 (40)
**Education**	No formal schooling	437 (18)	771 (28)	1208 (24)
	Less than primary school	148 (6)	133 (5)	281 (6)
	Primary school completed	469 (20)	518 (19)	987 (19)
	Secondary school completed	374 (16)	386 (14)	760 (15)
	High school completed	701 (29)	672 (24)	1373 (27)
	College/University completed	193 (8)	155 (6)	348 (7)
	Post graduate degree	59 (3)	111 (4)	170 (3)
**Social Group**	SC	891 (37)	1036 (38)	1927 (38)
	OBC/others	337 (14)	362 (13)	699 (14)
	General	1107 (47)	1303 (48)	2410 (47)
	Refused	45 (2)	46 (1)	91 (1)
**Marital Status**	Never married	532 (22)	306 (11)	838 (16)
	Currently married	1743 (73)	2132 (78)	3875 (76)
	Separated	20 (1)	17 (1)	37 (1)
	Divorced	14 (1)	11 (0)	25 (1)
	Widowed	55 (2)	263 (10)	318 (6)
	Refused	17 (1)	18 (1)	35 (0)
**Occupation**	Government employee	116 (5)	71 (3)	187 (4)
	Non-government employee	744 (31)	213 (8)	957 (19)
	Self-employed	1096 (46)	194 (7)	1290 (25)
	Student	145 (7)	162 (6)	307 (6)
	Homemaker	82 (3)	1985 (72)	2067 (40)
	Retired	89 (4)	25 (1)	114 (2)
	Unemployed (able to work)	73 (3)	69 (3)	142 (3)
	Unemployed (unable to work)	32 (1)	22 (1)	54 (1)
**Total**		2381 (46)	2746 (54)	5127 (100)

*Figures in parenthesis indicate percentages, Abbreviations: SC: Scheduled Caste, OBC: Other Backward Castes

Prevalence of current tobacco use (smoking as well as smokeless form) was 11.3% (95% CI: 9.3–13.3) (Table 2). More men (13.1%) smoked than women (0.3%). Overall current tobacco smoking (7.2%, 95% CI: 5.5–8.9) was more prevalent than smokeless tobacco consumption (5.2%; 95% CI: 3.8–6.5). Prevalence of tobacco smoking was high among adults aged 18–44 years old (8.0%, 95% CI: 6.1–10.0) and in urban areas (9%; 95% CI: 5.8–12.1) compared to those in rural areas (6.0%; 95% CI: 4.2–7.8).

**Table 2:** Prevalence of Various NCD Risk Factors in Punjab State, Overall and Stratified by Age Group, Sex, and Residence (Rural/Urban), 2014–2015.

	Behavioural Risk Factors (%, 95% CI)				
		Current tobacco users	Current drinkers	<5 servings of fruits and vegetables	Low physical activity
Age	18–44	11.2 (8.9–13.5)	13.6 (11.8–15.4)	95.4 (94.6–96.9)	32.3 (28.3–36.2)
	45–69	11.6 (8.8–14.4)	17.6 (14.4–20.8)	96.6 (95.2–98.0)	28.8 (22.5–35.0)
Sex	Male	20.1 (16.7–23.4)	27.4 (24.6–30.1)	95.3 (93.9–96.8)	22.4 (18.21–26.5)
	Female	0.9 (0.4–1.5)	0.3 (0.1–0.5)	96.4 (94.9–97.9)	41.3 (36.3–46.3)
Residence	Rural	11.2 (8.5–13.8)	14.9 (12.3–17.5)	96.4 (95.1–97.6)	32.6 (26.3–38.8)
	Urban	11.5 (8.3–14.7)	14.9 (12.9–16.9)	95.3 (93.1–97.5)	29.2 (28.1–35.3)
Overall		11.3 (9.3–13.3)	14.9 (13.2–16.6)	95.8 (94.6–97.0)	31.1 (26.7–35.5)
	Physical Measurements (%, 95% CI)				
		Overweight (BMI = 25.5–29)	Obesity (BMI≥30)	Abdominal Obesity (Males≥90, Females≥80)	Elevated Blood Pressure (SBP≥140 and/or DBP≥90 or currently on medication)
Age	18–44	25.0 (22.7–27.4)	10.7 (8.6–12.8)	M = 42.5(38.1–46.9)	30.4 (26.8–34.1)
				F = 60.8 (57.4–64.2)	
	45–69	36.2 (32.6–39.8)	17.3 (14.5–20.2)	M = 65.3 (59.3–71.4)	60.6 (57.2–64.0)
				F = 85.6 (81.7–89.5)	
Sex	Male	27.5 (24.3–30.7)	11.3 (8.9–13.7)	49.5 (45.3–53.7)	47.4 (42.7–52.1)
	Female	29.9 (27.1–32.7)	14.6 (12.5–16.7)	69.3 (66.5–72.0)	31.5 (28.7–34.4)
Residence	Rural	27.8 (25.1–30.5)	11.4 (9.7–13.2)	M = 48.5 (44.1–53.4)	41.1 (37.2–45.0)
				F = 66.8 (64.0–69.7)	
	Urban	29.7 (25.4–34.0)	14.7 (11.8–17.5)	M = 50.4 (42.7–58.1)	38.8 (34.6–42.9)
				F = 72.9 (67.6–78.1)	
Overall		28.6 (26.3–30.9)	12.8 (11.2–14.4)	-------------------------	40.1 (37.3–43.0)
	Biological Risk Factors (%, 95% CI)				
		Hyperglycemia (>110mg/dl)	Hypertriglyceridemia (>150mg/dl)	Hypercholesterolemia (>190mg/dl)	Raised salt intake (>5gm/day)
Age	18–44	8.4 (6.0–10.7)	19.8 (16.3–23.4)	15.2 (11.8–18.5)	86.4 (83.0–89.8
	45–69	27.7 (21.5–33.9)	25.6 (20.5–30.8)	18.3 (13.1–23.5)	87.7 (83.0–92.5)
Sex	Male	14.0 (10.7–17.3)	22.3 (17.4–27.3)	16.9 (12.7–21.1)	90.7 (87.1–94.3)
	Female	14.6 (11.1–18.2)	20.6 (16.2–25.0)	15.1 (12.4–17.8)	81.2 (77.1–85.2)
Residence	Rural	14.0 (10.1–17.9)	22.5 (18.2–26.9)	16.2 (12.5–20.0)	85.7 (81.6–89.9)
	Urban	14.6 (11.7–17.5)	20.3 (14.3–26.4)	16.0 (10.8–21.2)	88.3 (84.2–92.4)
Overall		14.3 (11.7–16.8)	21.6 (18.1–25.1)	16.1 (13.1–19.2)	86.8 (83.9–89.7)

Estimates are weighted for age, sex, and non-response on the basis of the population of State of Punjab in 2011

Prevalence of current use (last 30 days) of alcohol in the state was 14.9% (95% CI: 13.2–16.6) without any significant rural urban difference with about 27% (95% CI: 24.6–30.1) current users being men (Table 2). 36% of men drank alcohol in last 1 year. Harmful alcohol consumption (i.e. ≥ 60 g of pure alcohol for men and ≥ 40 g of pure alcohol for women on an average day) was found in 5% (95% CI: 3.7–6.2) of the population; significantly higher among men (9.5%; 95% CI: 6.7–11.3) than women 0.1% (95% CI: 0.0–0.2). Among current drinkers, one fourth consumed alcohol daily.

Low levels of fruits and vegetables intake was found to be high among both age groups, both sexes as well as residence. Overall 95.8% (95% CI: 94.6–97.0) of participants took less than 5 servings of fruits and/or vegetables on average per day. In a typical week, fruits and vegetables were consumed on 2.5 and 5 days respectively. 12.8% of the population (95% CI: 10.5–15.2) always/often added salt before/when eating.

Low levels of physical activity i.e. activity levels of less than 600 MET-minutes per week were prevalent among 31% (95% CI 26.7–35.5) of the participants (Table 2). Men (22.4%; 95% CI: 18.21–26.5) had a lower prevalence of low physical activity than women (41.4%; 95% CI 36.3–46.3). Both rural (33%) as well as urban (29.2%) areas had people with low physical activity. (Table 2)

Overweight and obesity was observed in 28.6% (95% CI 26.3–30.9) and 12.8% (95% CI: 11.2–14.4) of participants, respectively. (Table 2). The 45–69 years age group had significantly higher number of people having overweight (36.2%; 95% CI: 32.6–39.8) as well as obesity (17.3%; 95% CI: 14.5–20.2). The central obesity was found to be higher among women (69.3%; 95% CI: 66.5–72.0) than men (49.5%; 95% CI: 45.3–53.7).

The prevalence of hypertension (including those on medication for hypertension) was 40.1% (95% CI: 37.3–43.0). Significantly higher prevalence was observed among men (47%; 95% CI: 42.7–52.1), as compared to women (31.5%: 95% CI: 28.7–34.4).

In biochemical risk factors sections of Table 2, percentage of participants with values higher than cut offs specific to different parameters is presented. It was found that 14% (95% CI: 11.7–16.8) of the study participants had diabetes. The prevalence was higher among those aged 45–69 years (27.7%; 95% CI: 21.5–33.9). No difference was found in prevalence by sex and residence. Serum cholesterol and triglycerides were found higher than cutoff in 22% (95% CI: 18.1–25.1) and 16% (95% CI: 13.1–19.2) of the population. For both the parameters, values were higher for 45–69 years old, males and rural populations, although differences were not statistically significant.

Mean values of different parameters for behavioural information, physical measurements and biochemical assessments are presented in Tables 3 and 4. An average of 10 (95% CI: 8.0–11.6) cigarettes were being consumed per day by a daily smoker. Similarly mean number of standard drinks consumed per day by current drinkers were 2.3 (95% CI: 1.9–2.8). The mean value of fasting glucose level was 96.9 (94.2–99.6) with no significant difference found between men and women (Table 4). Mean cholesterol levels and triglycerides levels in the participants were found to be 149.8 (95% CI: 145.5–154.1) and 121.4 (95% CI: 115.7–127.1). Mean salt intake per day was found to higher for 87% (95% CI: 83.9–89.7) of population. It is interesting to note that out of 54% who said that they feel, they consume just the right amount of salt, had daily intake of salt more than 5gm/day. Mean salt intake in grams per day for the population was found to be 7.4 (95% CI: 7.2–7.7). Comparison between the different sub groups for mean values of the different biochemical parameters did not reveal statistically significant differences except between age and fasting glucose levels.

**Table 3:** Means (confidence intervals) of different parameters for behavioural information and physical measurements in population of Punjab State, 2014–2015.

	Mean of different behavioural risk factors on average per day, Mean (95% CI)					
	Mean amount of tobacco used by daily smokers on average per day	Mean number of standard drinks consumed on average per day	Mean number of servings of fruits and/or vegetable on average per day	Mean number of servings of fruits on average per day	Mean minutes spent in sedentary activities on average per day	Mean minutes of total physical activity on average per day
Age						
18–44	8.4 (6.4–10.4)	2.2(1.7–2.7)	2.3 (2.2–2.4)	0.7 (0.7–0.8)	221.4 (210.5–232.3)	230.3(211.4–249.2)
45–69	13.0(8.5–17.6)	2.6(1.9–3.3)	2.1 (2.0–2.2)	0.7 (0.6–0.8)	220.2 (220.2–261.3)	236.6(210.5–262.7)
Sex						
Male	9.7(7.8–11.5)	2.4(1.9–2.8)	2.3 (2.2–2.5)	0.7 (0.7–0.8)	218 (211–225)	280.5(257.7–303.2)
Female	11.6(0.6–22.5)	1.7(0.0–3.8)	2.1 (2.0–2.2)	0.7 (0.6–0.8)	225.1 (219–231)	175.3(155.1–195.5)
Residence						
Rural	8.7(6.4–11.0)	2.8(2.2–3.5)	2.2 (2.1–2.3)	0.6 (0.5–0.7)	222.1 (205.9–237.3)	229.4(202.6–256.1)
Urban	11.1(8.8–13.4)	1.7(1.2–2.1)	2.3 (2.1–2.4)	0.8 (0.7–1.0)	236.0 (218.1–253.1)	236.3(209.1–263.5)
Overall	9.8(8.0–11.6)	2.3(1.9–2.8)	2.2 (2.1–2.3)	0.7 (0.6–0.8)	228.0 (215.9–239.2)	232.3(213.3–251.4)
	Mean of different physical parameters, Mean (95% CI)					
	Height	Weight	BMI	WC	SBP	DBP
Age						
18–44	163.1 (162.7–163.6)	63.2 (62.1–64.2)	23.6(23.3–24.0)	85.7 (84.9–86.6)	125.7(124.6–126.8)	83.1(82.3–84.0)
45–69	161.2 (160.5–161.9)	66.8 (65.4–68.3)	25.8(25.3–26.3)	94.3 (93.0–95.7)	140.2(138.1–142.3)	88.7(87.5–89.9)
Sex						
Male	168.6(168.1–169.1)	68.4(66.9–69.9)	24.0(23.6–24.4)	89.5(88.4–90.7)	134.6(133.0–136.1)	87.2(86.4–88.1)
Female	155.2(154.9–155.6)	59.4(58.5–60.3)	24.7(24.4–25.0)	87.3(86.4–88.2)	125.4(124.2–126.7)	82.2(81.4–83.0)
Residence						
Rural	162.7(162.1–163.3)	63.4(62.5–64.4)	23.9(23.7–24.2)	87.7(86.9–88.5)	131.3(129.8–132.8)	85.3(84.2–86.4)
Urban	162.2(161.6–162.8)	65.4(63.6–67.3)	24.9(24.2–25.5)	89.6(88.0–91.2)	129.1(127.7–130.6)	84.4(83.5–85.4)
Overall	168.6 (168.1–169.1)	155.2 (154.9–155.6)	24.3(24.0–24.7)	88.5 (87.7–89.3)	130.4(129.3–131.5)	84.9(84.2–85.7)

**Table 4:** Means (confidence intervals) of different biochemical parameters in population of Punjab State, 2014–2015.

		Glucose	Triglycerides	Cholesterol	Mean Salt intake	Mean urinary Sodium
Age	18–44	91.7 (89.7–93.7)	118.8 (112.7–125.0)	147.2 (142.8–151.6)	7.3 (7.0–7.6)	105.8 (97.5–114.0)
	45–69	108.8 (102.3–115.3)	127.2 (119.4–134.9)	155.7 (149.2–162.2)	7.8 (7.7–8.4)	100.3 (92.1–108.4)
Sex	Male	96.4 (92.5–100.3)	122.6 (114.2–130.9)	150.6 (144.9–156.4)	8.1 (7.7–8.4)	103.4 (95.6–111.3)
	Female	97.5 (94.9–100.2)	119.8 (113.0–126.7)	148.7 (145.1–152.3)	6.7 (6.4–6.8)	105.0 (95.4–114.6)
Residence	Rural	96.5 (92.4–100.6)	122.3 (116.6–128.0)	151.6 (147.3–156.0)	7.8 (6.9–7.6)	99.4 (90.3–108.4)
	Urban	97.5 (94.2–100.8)	120.2 (109.1–131.3)	147.3 (138.8–155.7)	7.7 (7.2–8.2)	110.6 (99.3–121.8)
Overall		96.9 (94.2–99.6)	121.4 (115.7–127.1)	149.8 (145.5–154.1)	7.4 (7.2–7.7)	104.1 (96.0–111.0)

In the assessment of past history of hypertension and diabetes, 17.6% and 5.7% of participants reported a positive history. Out of these, 84% of hypertensive participants were on medication. For diabetes, the corresponding proportion was 58%. Interestingly more than one-third of the population (37%) had never previously got for their blood pressure measured. Similarly, a positive history of cardiovascular disease (i.e. ever had a heart attack or chest pain from heart disease (angina) or a stroke (cerebrovascular accident or incident) was reported by 4.4% of the participants. Only 1.5% of participants with cardiovascular disease were on preventive medication (aspirin/statins).

**Fig 1:**
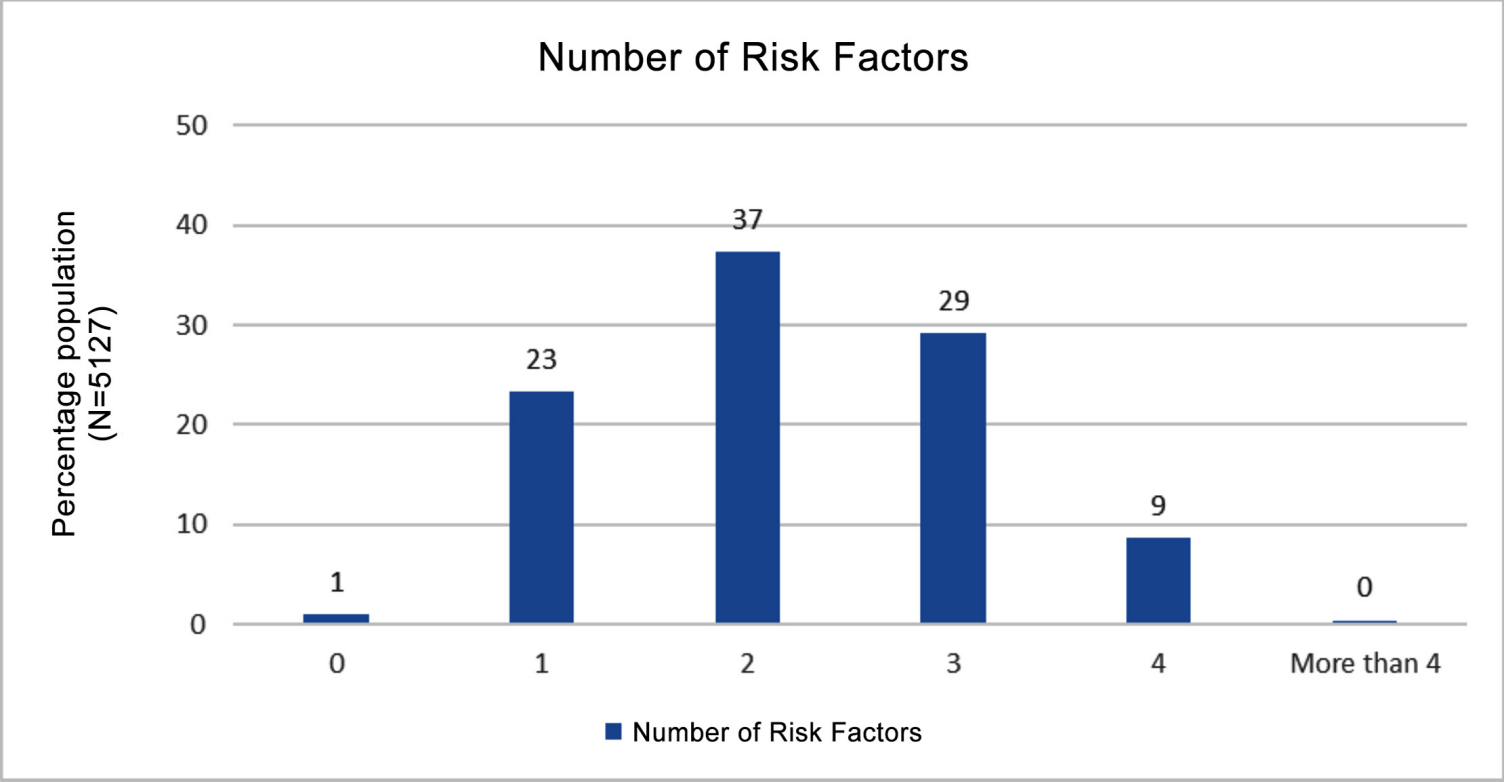
Percentage of participants with number of high risk factors for NCDs.

More than half of the participants (60%) had at least one risk factor, one third had 3–6 risk factors. Only 1% (95% CI: 0.7–1.7) of the population was completely free from the 6 established risk factors (i.e. current smoking, harmful alcohol use, less than five servings of fruits and vegetables per day, not meeting WHO recommendations on physical activity for health (<150 minutes of moderate activity per week, or equivalent), overweight or obese (BMI ≥ 25 kg/m2), raised BP (SBP ≥ 140 and/or DBP ≥ 90 mmHg or currently on medication for raised BP) (Fig 1). In addition, 7% of the population aged 40–69 years had a cardiovascular risk of ≥ 30% over a period of next 10 years.

Utilization of preventive services for different cancers was low; only 4% and 2% of women aged 30–49 were ever screened for cervical and breast cancer respectively, whereas 7.9% (95% CI: 6.3–9.5) participants reported to have been screened for oral cancer.

Results of multiple logistic regression analyses (Table 5) highlight the increasing prevalence of different risk factors with age, adjusting for other factors including sex and the place of residence. As the education level increased, odds of being a smoker or alcoholic decreased significantly as compared to those with no formal schooling. However, with increase in education levels, odds of being less physically active increased, increase in blood glucose and cholesterol levels. Those belonging to Scheduled Castes, had higher odds of being smoker (Odds Ratio: 2.3, 95% CI: 1.7–3.1). Women were found to be having significantly higher odds of obesity (Odds Ratio: 1.2, 95% CI: 0.9–1.6) and hypercholesterolemia (Odds Ratio: 1.4, 95% CI: 1.0–2.0) but lower odds of having hypertension, adjusting for other socio-demographic characteristics. Compared to participants in rural areas, urban area participants had significantly higher odds of low physical activity levels (Odds Ratio: 1.5, 95% CI: 1.3–1.8), obesity (Odds Ratio: 1.2, 95% CI: 1.0–1.4) and hyperglycemia (Odds Ratio: 1.2, 95% CI: 1.0–1.4).

**Table 5:** Determinants of NCD risk factors among aged 18–69 years in the State of Punjab, 2014–2015.

		Current SmokersOR(95% CI)	Current DrinkersOR(95% CI)	Physical ActivityOR(95% CI)	Insufficient servingsOR(95% CI)	Raised Blood PressureOR(95% CI)	ObesityOR(95% CI)	Raised GlucoseOR(95% CI)	Raised CholesterolOR(95% CI)
Education	No formal Schooling	1	1	1	1	1	1	1	1
	Primary Education	0.8 (0.6–1.1)	0.9 (0.7–1.1)	0.6* (0.5–0.8)	0.4* (0.2–0.7)	1.1 (0.9–1.3)	1.6* (1.2–2.0)	1.5* (1.1–1.9)	1.9* (1.4–2.8)
	Secondary Education	0.5* (0.3–0.7)	0.6* (0.5–0.9)	0.7* (0.6–0.9)	0.3* (0.2–0.7)	1.0 (0.8–1.2)	1.7* (1.3–2.3)	1.2 (0.8–1.7)	2.2* (1.5–3.3)
	Higher Education	0.3* (0.2–0.4)	0.6* (0.4–0.7)	0.9 (0.7–1.1)	0.2* (0.1–0.4)	1.0 (0.8–1.2)	1.6* (1.3–2.1)	1.5* (1.1–1.9)	2.4* (1.7–3.4)
Social Group	General	1	1	1	1	1	1	1	1
	Scheduled Caste	2.3* (1.7–3.1)	1.1 (0.9–1.3)	0.7* (0.6–0.9)	1.3 (0.9–1.8)	0.8 (0.7–1.0)	0.8* (0.6–0.9)	1.0 (0.8–1.2)	0.8 (0.6–1.1)
	Other Backward	1.4 (0.9–2.0)	1.0 (0.8–1.3)	0.6* (0.5–0.8)	1.0 (0.7–1.6)	0.8* (0.7–1.0)	0.9 (0.7–1.2)	1.2 (0.9–1.6)	1.1 (0.8–1.5)
Gender	Men	1	1	1	1	1	1	1	1
	Women	0.0* (0.0–0.1)	0.0* (0.0–0.0)	0.8* (0.6–1.0)	1.0 (0.7–1.5)	0.6* (0.5–0.7)	1.2 (0.9–1.6)	0.9 (0.6–1.2)	1.4 (1.0–2.0)
Age group	18–44	1	1	1	1	1	1	1	1
	45–69	0.9 (0.7–1.2)	1.0 (0.9–1.2)	1.2 (1.0–1.4)	1.0 (0.7–1.4)	2.8* (2.5–3.2)	1.4* (1.2–1.7)	3.5* (2.8–4.3)	2.1* (1.6–2.6)
Marital Status	Currently married	1	1	1	1	1	1	1	1
	Never married	1.4 (1.0–1.9)	2.8* (2.2–3.6)	1.3 (0.9–1.7)	1.0 (0.6–1.4)	2.1* (1.8–2.6)	4.0* (2.7–6.2)	3.0* (1.9–5.0)	2.5* (1.6–4.1)
	Divorced/ Widowed	1.3 (0.7–2.5)	3.4* (2.1–5.5)	0.9 (0.7–1.1)	1.0 (0.7–1.4)	3.1* (2.3–4.1)	4.5* (2.7–7.4)	3.7* (2.1–6.6)	3.1* (1.7–5.6)
Residence	Rural	1	1	1	1	1	1	1	1
	Urban	1.7* (1.3–2.2)	1.0 (0.8–1.2)	1.5* (1.3–1.8)	0.7* (0.5–1.0)	0.9 (0.8–1.1)	1.2* (1.0–1.4)	1.2 (1.0–1.4)	1.0 (0.8–1.3)
Occupation	Home Maker	1	1	1	1	1	1	1	1
	Government Employee	5.3* (1.7–17.1)	15.7* (5.7–43.9)	0.9 (0.7–1.2)	0.3* (0.2–0.7)	1.0 (0.7–1.5)	0.8 (0.5–1.3)	0.7 (0.4–1.1)	1.9 (1.1–3.1)
	Non-Govt. Employee	4.2* (1.4–12.0)	17.0* (6.5–44.9)	1.0 (0.7–1.5)	0.6* (0.3–1.0)	0.8* (0.6–1.0)	0.4* (0.3–0.6)	0.5* (0.3–0.7)	0.6* (0.3–0.9)
	SelfEmployed	2.6 (0.9–7.7)	16.2* (6.2–42.7)	1.1 (0.9–1.5)	0.7 (0.4–1.1)	1.1 (0.9–1.4)	0.8 (0.6–1.1)	0.6* (0.4–0.9)	1.3 (0.9–2.0)
	Others	2.9 (0.9–8.7)	13.1* (4.9–35.1)	1.1 (0.9–1.4)	0.5* (0.3–0.8)	1.8 (0.8–1.3)	0.7 (0.5–1.0)	0.7 (0.5–1.1)	1.1(0.7–1.7)

*Significant Results: P value <0.05; OR = Odds Ratio; CI = Confidence Interval

## Discussion

This survey which assessed the prevalence of multiple risk factors for NCDs is first ever STEPS survey conducted in Punjab State, and the first in last decade in the country. This study demonstrated that the Punjab has a particularly high prevalence of NCD risk factors which are comparable to several developed countries. Risk factors were present uniformly without any significant variation in prevalence by age group, gender or place of residence.

Our findings of tobacco use (smoking as well as smokeless form) are consistent with the Global Adult Tobacco Survey for Punjab State, District Level Household and Facility Survey for state.[5, 6] The prevalence is low in comparison to neighboring states [5, 12] and national average of India (15%)[12] as 58% of the state’s population belongs to Sikh religion, which sanctions against tobacco use.[17, 18] Consumption was higher among men than women, which could be due to the social unacceptability of women’s use of tobacco in the state.

Findings related to prevalence as well as patterns of alcohol use in the state were similar to familiar patterns in literature.[6, 12, 19, 20] Harmful use of alcohol, prevalent in about 5% (95% CI: 3.7–6.2) of the total study population, is similar to those reported by other STEPS Surveys.[20, 21] As in our survey, Wilsnack et al, had also found a higher prevalence of alcohol use (especially harmful use) among older age groups and claimed contrary to belief that alcohol use is a problem among reckless youth and prevalence declines as people mature and take responsibilities.[22] Patterns of alcohol use identified in the survey are detrimental to health of people of Punjab. In the absence of a public health driven alcohol policy, poor implementation of existing legislations, the problems due to alcohol use are bound to increase in the coming decades in the state.

It was found that the vast majority (96%) of the study population consumed less than the WHO recommended daily five servings [8] of fruits and vegetables. Findings may be similar to reports from other STEPS Surveys [20, 23, 24] but are unacceptable for a state considered as “food basket” of nation. As availability and accessibility are not major issue, this may be considered population’s dietary preference, probably due to underlying fear of ingestion of potentially contaminated produce due to pesticides. Punjab state has been reporting a rising trend in cancer cases and high pesticide use has been reported as one of the risk factor for the same.[25] Attitude and practices, therefore, need to be explored further to understand the underlying reasons. This finding is a serious concern and needs designing of appropriate public health interventions with the understanding of local concerns of population.

The current study findings on prevalence of low physical activity (31%) and participants reporting no vigorous activity (72%) point towards a growing epidemic of overweight and obesity, and should also be a major cause for concern for public health authorities. Since Punjab is predominantly an agriculture driven state, low physical activity levels are not as high as reported in other surveys in country.[12] In the rural sector, Punjab is facing an acute labour shortage following introduction of schemes by the central/state government to support livelihood in their native states. This could be one reason why in our survey, rural areas have higher level of high physical activity (26.2%) as compared to urban areas (23.1%). It could also reflect the relationship between a predominantly agrarian society in the rural areas and lower availability or reliance on motorized vehicles.

A high prevalence of overweight and obesity has been observed in this study, which is higher than several other states in the country. The results are comparable to Kerala state [12], a state fast moving towards advanced stages of epidemiologic transition.[12, 26] The higher prevalence of being overweight among urban residents is consistent with results from neighboring states and countries.[20, 23, 24, 27] Further the prevalence is comparable to obesity levels in many developed countries. [2]

High prevalence of hypertension (40%) in the state is alarming and adds to estimates from other cross sectional surveys in the state as well as surrounding regions.[6, 28, 29] In Global Burden of Disease study (Indian estimates), blood pressure was one of the three leading risk factors for national disease burden and the same is true for Punjab state as well. [30] Self-reporting of hypertension suggested high treatment rates for hypertension (84%), however, survey revealed a huge amount of undiagnosed hypertension. Keeping in account the newly diagnosed hypertensive population, 42% of participants with raised blood pressure were not currently under any treatment for raised blood pressure, which indicates a high unmet need for prevention and management.

The observed prevalence of diabetes in the current study was 14%, which is higher than estimated value for the surrounding states [31] and the country (9%) [2] and several countries of South East Asia [2] [20, 27, 32, 33]

In the current study, tobacco smoking, alcohol drinking and raised blood pressure (BP) were more frequent in males than females which is consistent with findings from several other STEPS Surveys. [20, 29, 31, 34, 35] However, low physical activity and obesity were more prevalent among women. This may reflect cultural norms and the greater role of women as homemakers, who are less likely to step outside for regular physical exercise, although this may be changing in the younger and more educated segments of the population.

Though prevalence estimates are important from the perspective of prevention and control measure of NCDs, mean values of indicators act as references and are important to gauge the effects of interventions. Mean body mass index, systolic/ diastolic blood pressure, fasting blood glucose and triglyceride levels in our study have been found to be similar to that reported by other studies, [31, 34] but mean total cholesterol levels were lower than earlier reported in literature.[24, 36–38] No recent population based study reporting mean levels of cholesterol lipoproteins and prevalence of various dyslipidemias in Punjab could be found.

The fact that only 1% of the study population was found to be free of any studied NCD risk factors is an indication of growing risk and potential epidemic of NCDs in the state which represents a huge public health challenge to health system.

Proportion of 40–69 year old adults with a 10-year risk of cardiovascular disease ≥30% was also substantial at 7.1%, which is higher than estimates for several countries in South East Asia region.[20, 21, 39] Considering the high burden, all individuals with an absolute CVD risk of ≥30% or more over 10 years should be targeted for comprehensive risk factor management, which may include, blood pressure and lipid lowering therapy and bring risk down to 15% absolute CVD risk. For individuals with absolute CVD risk less than 15% over the next 10 years, lifestyle modification is recommended.

The screening for common cancers in state is very low and needs improvement. Punjab Government recently completed a state wide campaign for early detection and awareness of cancer,[40] but efforts need to be focused for organized screening for common cancers.

Significant association was found between different NCD risk factors and demographic characteristics. Statistically significant association was found between education and tobacco use, physical activity & fruit, vegetable intake (P value: <0.05). Similar to findings of other studies tobacco use, alcohol consumption and low physical activity was significantly higher in men than women. A significant association was found between level of education and tobacco & alcohol consumption (P value: <0.01). Lower social group had significant association between tobacco consumption, and low physical activity (P value: <0.05). [41]

This study has a few limitations. It is a cross sectional study design which limits causality of relations. However, large sample size of study make the results conclusive. Second issue is of over reporting, which is a well-recognized issue for self-report surveys as participants tend to report in socially desirable ways.[42] For example, the less active may want to over-report activity to appear healthier. Use of range checks and cross matching of data with physical and biochemical parameters helped us to control this bias.

In India, NCD Risk factor surveys have been planned to be conducted under the Integrated Disease Surveillance project [43], as part of efforts to establish surveillance system for NCDs. However no such survey could be conducted at the national level in India. During one of the only sub national survey of 7 states (2007–08), Punjab State was not covered.[12] Conducting this survey in the state threw several challenges upon investigators in terms of low budget (only INR 3.8 million against recommended minimum of 9 million, till step 2), non-availability of personal digital assistants, high cost of cholesterol measuring strips. Challenges were overcome by use of mix of dry and wet chemistry, excellent coordination between health department & tertiary care public private medical institutes for temporary storage facilities and local supportive supervision. Active community involvement for stay arrangements and ensuring cooperation from participants led to successful completion of survey.

Overall, survey findings suggests that NCD risk factors are, in general, common and almost uniformly prevalent in the adult population of the Punjab state. The estimates generated by this survey provided baseline data for state wide action plan prepared by state for specific population and individual health interventions for implementation. It helped the state to adopt cardiovascular disease risk assessment and management under the NCD control program to lower the population at risk of CVDs. The use of STEPS methodology will enable future state-wide, national and international comparisons.

## Supporting Information

S1 Dataset Raw data of NCD Risk Factors Survey, Punjab 2015. (XLSX)
